# Looking beyond seeing: Components of visual-spatial ability as an overarching process

**DOI:** 10.1016/j.actpsy.2024.104577

**Published:** 2024-11

**Authors:** Moran Bar-Hen-Schweiger, Avishai Henik

**Affiliations:** a Department of Psychology, Ben-Gurion University of the Negev, Beer Sheva, Israel; b The Zelman Center for Brain Science, Ben-Gurion University of the Negev, Beer Sheva, Israel

**Keywords:** Visual-spatial, Transformation, Rotation, Perspective-taking, Mental manipulation, Intelligence

## Abstract

Visual-spatial skills have been a fertile ground for assessing aspects of intelligence and investigating its components. The initial goal of this bipartite study was to elucidate the nature of the underlying components of visual-spatial processes and the relations among them. The second goal was examination of a higher, overarching factor, underlying spatial ability but also lexical-semantic performance as well. In Study 1, three components model is examined, hypothesized to form the foundation for visual-spatial processing. In Study 2, we utilized the findings from Study 1 and performed a structural model analysis with the aim of examining the hypothesis of a second-order factor, underlying both visual-spatial and lexical-semantic processes. These studies were motivated by the notion that underlying such visual-spatial and lexical-semantic skills is a factor we termed mental manipulation, which is domain-general that cuts across species.

One hundred and thirty-three participants completed 9 tasks, representing visual-spatial and lexical-semantic abilities. Confirmatory factor analysis (CFA) of the resulting data was utilized to model the results and compare the fitness of one-, two- and three-factor models. After establishing the measurement model, a second-order structural model analysis was performed to assess the existence of an overarching factor, common to both verbal and visual domains. The results of the analyses confirm the existence of a second-order factor, which we regard as reflecting mental manipulation. The implication of such mental manipulation is discussed in terms of practical applications for diagnosis, intervention, and education, highlighting its potential to improve outcomes.

## Highlights

• CFA analyses resulted in a three-component model of visual-spatial ability.
• Second order factor was proposed as an overarching process, beyond the spatial domain.
• CFA and SEM analyses assessed different models, when verbal tests were added.
• The three-component model provided a good fit to the data after adding verbal tests.
• Second order model combining visual-spatial and verbal tests, described the data best.

## 1 Introduction

Inspired by a comparative development approach, studies of non-human cognition, and the work of Piaget (e.g., 1952, 1971), we propose a new concept of intelligence couched in a biological context. The evolution of intelligence is marked by a shift from concrete to abstract operations, or to the transition from object manipulation to mental manipulation (Bar-Hen-Schweiger & Henik, 2020; Bar-Hen-Schweiger et al., 2017; Bar-Hen-Schweiger & Henik, 2019; Henik et al., 2021). We suggest that the operations we refer to as essential to intelligence (e.g., abstract transformations, rotations, perspective-taking, and verbal tasks) are fundamentally acts of mental manipulations (MM). These authors' description of the evolutionary development of Object manipulation, refers to the direct physical handling and transformation of objects, allowing individuals to explore properties such as shape, size, and orientation. This sophistication of tool use across species serves as a foundation for mental manipulation. For example, motor actions are performed by animals or humans on objects in order to achieve desired goals, which may evolve into Mental Manipulation, referring to the cognitive process of mentally transforming, or operating on, perceived or imagined objects, without the need for physical interaction with actual objects. This includes tasks such as rotating objects in one's mind, solving abstract problems, comparing quantities or shapes, or visualizing changes in complex systems. An example of an early study of quantifying abstract mental manipulation, is the famous study of Shepard and Metzler (1971), who timed the mental movement of imagined object rotations. Another example of mental manipulation is perspective-taking, referred to as the ability to adopt a viewpoint of a scene different from one's own, or the ability to consider the consequences of an action before executing it. Note that mental manipulation is an overarching principle reflecting the ability to perform transformations, translations, re-combinations, projection, and prediction in infinite ways. and as an underlying building block of intelligence.

Object manipulation viewed as a set of processes, organized hierarchically and in developmental sequences, found across different species (Bard & Gardner, 1996; Gardner & Gardner, 1971; Parker, 1977). In the most complex forms of these processes, as noted above, are mental manipulation achieved by humans. Thus, even solving algebraic equations involves MM. This hierarchical transition suggests the existence of overarching concepts underlying cognitive activities across different domains. In the present study, we investigate such an underlying common MM in three general aspects of the visuospatial domain, using widely used visual-spatial tasks. After demonstrating the underlying MM factor common to them using structural equation modeling, we add two lexical-semantic tasks requiring abstraction, in order to investigate whether or not they share an underlying MM factor with the visual-spatial tasks.

Visual-spatial skills have been an integral modality both for assessing aspects of intelligence and for investigating its components. In humans, visual-spatial skills can be seen as being various forms of abstract object manipulations. Accordingly, research has conveyed evidence for the association between visual-spatial processing and intelligence, and batteries for assessing intelligence include visual-spatial tests. As such, it may be the case that latent factors, considered the building blocks of visual-spatial abilities, are actually overarching processes that are at the heart of our mental processing in other domains as well, such as emotion (Gilead et al., 2016), lexical-semantic processing (Greenfield, 1991) and the biological and continuous nature of intelligence, (Piaget, 1952, Piaget, 1964), which we regard as mental manipulation. Research on visual-spatial skills has suggested the existence of several latent factors, such as perspective-taking and mental rotation. However, since solving visual-spatial tasks may be approached with more than just one specific strategy, it seems that characterizing a spatial skill by using a single, primary operational terminology, such as rotation or perspective taking, is insufficient to account for individual differences in spatial abilities. Accordingly, we propose to add another underlying process to the two-factor model that we refer to as *transformation*. In the larger context, then, mental rotation, perspective-taking, and transformation can be seen as being different aspects of abstract object manipulation or mental manipulation. The current work is an attempt to sharpen our understanding of the underlying processes comprising the mental manipulations which underlie visual-spatial aptitude. Furthermore, we examine the existence of an overarching process of mental manipulations, reflected beyond the visual-spatial domain but also in lexical-semantic processes, which we suggest is just another form of mental manipulation.

To this end, we present two studies. The first one deals with visual-spatial tasks. The second examines whether tasks that are not visual-spatial in nature would map onto the same latent factors found in the visual-spatial domain, and the possibility that a higher-order factor could explain the relations among these factors. If this notion is supported by the data, these factors, traditionally considered to represent spatial skills, might potentially be implicated in wider aspects of human intelligent behavior.

### 1.1 Factors of spatial ability

In a field notable for many proposals for processes underlying visual-spatial abilities, there is wide agreement among investigators regarding at least one major factor, namely, spatial visualization. Spatial visualization has been defined, for example, as the “ability to manipulate or transform the image of spatial patterns into other arrangements” (Ekstrom et al., 1976, p. 173). Yet another common definition of spatial visualization, suggested by Carroll (1993), is “the power in solving increasingly difficult problems involving spatial forms” (p. 315). Spatial visualization tests involve rotation and transformation of two- or three-dimensional objects. This factor is typically assessed by performance on a range of tasks, such as paper folding and mental rotation (McGee, 1979). McGee, who reviewed psychometric studies of spatial capability, suggested one more basic factor: spatial orientation. He defined spatial orientation as “the comprehension of the arrangement of elements within a visual stimulus pattern and the aptitude to remain unconfused by the changing orientation in which a spatial configuration may be presented” (p. 893). This spatial orientation factor was also termed *perspective taking*, since it involves the ability to imagine an object from different perspectives with respect to the observer's point of view.

Lohman (1979) re-analyzed psychometric studies of spatial ability and identified yet a third spatial factor, in addition to the two mentioned above; namely, *spatial relation*, referring to rapid performance during simple tests such as card rotation (Ekstrom et al., 1976). Compounding the complexity of an already crowded arena, a five-factor theory was proposed by Carroll (1993): visualization, spatial relations, closure speed, closure flexibility, and perceptual speed. However, Carroll's five-factor approach was not fully supported by further studies, since two factors—closure flexibility and closure speed—did not show consistent evidence of being separable. Interestingly, several researchers reported high correlations between spatial visualization and spatial orientation factors (Carroll, 1993; Hegarty & Waller, 2005; Lohman, 1979, Lohman, 1988). Such high correlations raise doubts as to the uniqueness and differentiation of visual-spatial aptitude into these two factors (i.e., spatial visualization and spatial orientation). Moreover, Lohman (1979) also concluded that spatial orientation tests are often solved by mental rotation strategies, such as object manipulation, which makes the spatial orientation factor more difficult to differentiate from other spatial factors.

### 1.2 What creates inconsistencies in spatial factors?

As can be gathered from the foregoing brief review, there has been lack of coherence in describing the underlying cognitive processes forming the foundations of visual-spatial skills.

Perhaps a succinct statement of this variability problem in the field was expressed by Guttman et al. (1990). Responding to the inconsistency in the arena of factor-analytic studies described above, they proposed an alternative approach to conceptualization of visual capabilities, involving facet theory and regionality. For this purpose, they reviewed the literature on factorial studies of classifying spatial abilities, and summarized the issue thusly: “Certain factor names, such as visualization and orientation, reappear in several different studies, but the various authors have not been consistent in assigning the same definitions to the same names (p. 220)”.

Today, these issues persist; for example, the utilization of tests with similar titles or descriptions being assigned to different features or instructions (Lohman, 1979; Pellegrino et al., 1984). Although measurement issues (“pureness” of the skill to be measured by the task) are not limited to visual-spatial studies, this issue has important implications for our understanding of the essential components of spatial abilities. Tests usually comprise several processes in addition to the skill being measured, such as task characteristics, perhaps even other, non-relevant, cognitive processes, as well as measurement error. Therefore, the results may not be consistent and could confound the proposed models of visual abilities per se. However, pureness of measurements can improve through a careful analysis of the commonalities within and between tests and, certainly, by careful choice of tasks and replications of results.

### 1.3 Transformation: A common underlying process

In light of the foregoing brief review, we propose another underlying process—*transformation*—that should be added to the perspective-taking (also known as spatial orientation) and mental rotation factors proposed by Kozhevnikov and Hegarty (2001), and Hegarty and Waller (2004). It seems that referring to spatial skills by using rotation or orientation as primarily operational terminology is insufficient to account for individual differences in spatial abilities (Kosslyn, 1981; Kosslyn et al., 1984; Kosslyn et al., 2006). The proposed factor, or dimension, refers, therefore, to another level of visual-spatial task performance, which involves examination and change (shaping, molding, matching) of mental representations, and consequently, it is reasonable to consider it as a separate factor. Transformation refers here to common visual-spatial operations, which seem to be involved in most, if not in all, spatial processes. This process takes place during different stages of performance and may serve as a common ground, binding between the specific operations, such as rotation or perspective taking. Moreover, we can perform different types of changes, modifications, and re-combinations on concrete and abstract entities (e.g., depictions, ideas, statements) in our minds. Accordingly, transformation refers to more than just one dominant strategy for changing an object, such as rotation or changing perspectives, since different visual-spatial tasks may be handled by more than just one specific strategy. Thus, we regard transformation as the evocation of the mental image of a task, which provides information for either possible solutions or material for further operations. In addition, this proposed common process, which we assume to take place at different stages across spatial tasks, might be another explanation for the strong correlations among spatial components. This is so since some tests require a sequence of spatial operations accompanied by this common stage, whereas others may be completed without performing any additional procedures. Despite our prediction that transformation is a separate spatial factor, it is likely to be involved in a variety of other cognitive processes (e.g., translation of an abstract goal into specific sub-goals by mentally contemplating a sequence of actions), including tests that traditionally have been classified under spatial and even non-spatial factors. The three components (transformation, perspective and rotation), then, could possibly reflect a building block comprising human intelligence, characterized by the ability to perform mental manipulations.

### 1.4 The current study

Two studies were designed to explore the underlying nature of visual-spatial processes. The current (first) study is divided into two stages, with the first stage providing data for use in the second stage. Specifically, the first stage specifies the measurement model, that is, assigning tasks to the factor they are assumed to tap, or represent. Clarifying the commonality and uniqueness processes assessed by different types of spatial measures enables us to address the structural relationships among the factors in the second stage. The structural model analysis estimates the measurement model and the structural relationships among the factors, combining the two in one model. A second-order model contains two layers of factors—measurement and structural—with causal paths from the second-order factor to the first-order factors (transformation, perspective taking and rotation). Therefore, the structural model is more parsimonious compared to the measurement model. The second-order model approach can provide theoretical interpretation of the significant correlations, if any, among the first-order factors. It may appear to capture some aspects of spatial ability, but might also represent more general, overarching aspects of performance. That is, by using structural analysis, we can test whether a higher factor, which may embody a more general component compared to the first layer factors, and examine the commonality among the three first-order factors. Namely, we propose that the three components (transformation, perspective and rotation), then, could possibly reflect a building block comprising human intelligence, characterized by the ability to perform mental manipulations.

In the first study, the goal was to assess the distinction between transformation, perspective-taking and rotation factors. Note that our model was developed in the spirit of Hegarty and Waller's (2004) two-factor model. Hegarty and Waller found a close relation between mental rotation and orientation/perspective-taking factors (*r* = 0.81), but still considered them as representing distinct spatial components. However, the correlation between these two factors in Hegarty and Waller's model is rather high and may raise questions regarding the dissociation/association of these two factors. However, we hypothesize that a three-factor model describes the underlying structure better, and each factor describes a different aspect of spatial performance, and we expect a better fit and possibly higher loadings of tasks on their respective factor.

Given the high correlations among visual-spatial factors in previous studies, we expected to find correlations among these latent variables. Moreover, we first hypothesize that a possible reason for this consistent finding is that these visual-spatial factors, which traditionally have been considered as spatial factors, are involved in non-spatial tasks. Thus, the commonality among the factors should enable us to assess our second hypothesis, namely, that there are overarching processes that are shared by and carried out across all tasks. In Study 2, we examined the possibility that a higher factor, domain-general in nature, is involved in different types of skills, extending beyond visual-spatial abilities.

To summarize, in the present study, we examine the following two hypotheses, each follows from the findings of the previous one:

1) A three-factor model describes the underlying structure of visual-spatial performance better than a two-factor model.
2) An underlying second-order ‘general’ factor (perhaps representing intelligence) is hypothesized to be common to visual-spatial and lexical-semantic tasks, such that it reflects more general cognitive processes of mental manipulations.

## 2 Study 1

### 2.1 Method

#### 2.1.1 Task selection

We selected a group of tasks commonly used in the field of cognitive visual-spatial research, to represent closely the range of these abilities (Table 1). Therefore, for the perspective-taking factors, the chosen tasks were the Cross Section Test (Cohen & Hegarty, 2012), the Object Perspective Test/Perspective Taking (Hegarty & Waller, 2004; Kozhevnikov & Hegarty, 2001) and the Visual-Spatial Test (NeuroTrax Corp., Texas). The object-reference tasks assumed to load on the rotation factor were the Mental Rotation Test (Ganis & Kievit, 2015) and the Revised Purdue Spatial Visualization Test: Visualization of Rotations (Revised PSVT: R, Yoon, 2011). As described above, the transformation factor is presumed to reflect primary processes, such as, for example, image formation, which probably entails more than just one specific act. Accordingly, we selected tasks that seemed to be solvable by using a mental alteration and comparison of a visual-spatial image. The tasks assumed to tap the transformation factors were the Mental Paper Folding Test (MPFT; Wright et al., 2008) and the Mental Clock Test (Paivio, 1978).

**Table 1:** Latent factors and tests used.

Latent factors	Measured variables
Perspective factor	• Cross Section Test (Cohen & Hegarty, 2012) • Object Perspective Test/Perspective Taking (Hegarty & Waller, 2004; Kozhevnikov & Hegarty, 2001) • Visual-Spatial Test (NeuroTrax Corp., Texas)
Rotation factor	• Mental Rotation Test (Ganis & Kievit, 2015) • Revised Purdue Spatial Visualization Test: Visualization of Rotations (Yoon, 2011)
Transformation factor	• Mental Paper Folding Test (Wright et al., 2008) • Mental Clock Test (Paivio, 1978)

#### 2.1.2 Participants

One hundred and thirty-three undergraduate students (Mean age = 24.34 years, *SD* = 2.07, 103 females), recruited from the Experiment Subject Pool at Ben-Gurion University of the Negev, Israel, took part in the study. Our proposed model is not considered to be extremely complex (three latent variables), and keeping in mind that our sample did not contain missing data, and we used a maximum-likelihood estimation technique, it seems that our model can be adequately estimated with our sample size (Hair et al., 2009).

Participants were compensated with class credit or 70 NIS (approximately $18 US). All participants reported normal or corrected-to-normal vision, had no history of attention deficit disorder or a learning disability, and were native Hebrew speakers. All participants provided written informed consent prior to the commencement of the procedure.

#### 2.1.3 Materials

The participants completed the following seven computerized tests, which were programmed using *E*-prime version 2.0.10.356 software (Psychology Software Tools, Pittsburgh, PA). Dataset: https://data.mendeley.com/datasets/kh5zvysyph/1.

##### 2.1.3.1 The revised purdue spatial visualization test: Visualization of rotations (revised PSVT: R; Yoon, 2011)

We used a computerized version of the revised version of the Purdue Spatial Visualization Test – Rotation (Guay et al., 1976). The test measures the ability to visualize the mental rotation of a three-dimensional object. In each trial, participants were shown a slide that had a criterion object on the top part of the slide and a rotated version of the same object next to it. A second object, the target, was shown in the middle of the same slide. Participants were required to mentally rotate the target object in the same manner as the criterion object was rotated. Participants were instructed to select the correct object out of five options, given at the bottom of the slide, which showed the target object after it had been rotated in the same manner as the criterion object above it.

The test consisted of 30 multiple-choice problems, which contained 13 symmetrical and 17 nonsymmetrical drawings of three-dimensional objects, drawn in a three-dimensional format. Participants were given two practice trials before the actual test. Each trial began with a fixation point “+” for 500 ms prior to the onset of the problem slide. The slide remained on the screen until the participants responded. Response accuracy and reaction times were recorded.

##### 2.1.3.2 Mental rotation test

**Fig. A2:**
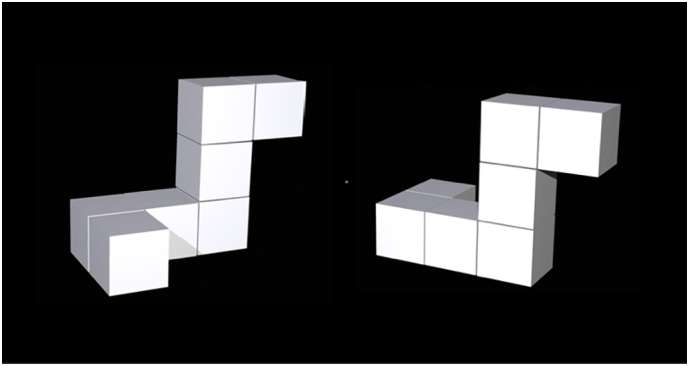
Example of an item from the Mental Rotation Tests (Ganis & Kievit, 2015).

We used a computerized version of the Shepard and Metzler (1971) Mental Rotation Test with stimuli from Ganis and Kievit's (2015) database (for validation data and stimuli please see: doi:https://doi.org/10.6084/m9.figshare.1045385) In each trial, participants were presented a pair of three-dimensional images of objects. In some pairs, the second image was a rotated version of the first, whereas in others, the second was a rotated mirror image of the first (see Fig. A2). The rotational angles of the pairs were presented in one of four possible orientations, from 00 (no rotation) to 150° (i.e., 00°, 50°, 100°, 150°). Participants were required, for each pair of images, to decide whether the two objects were identical (‘same’) or different (mirror images). The participants completed five practice trials, followed by four blocks of 40 trials each (160 trials in total). Each trial began with a fixation point “+” for 1500 ms and was followed by a blank slide for 200 ms. Then, the stimuli appeared and remained on the screen until the participant made a response. Accuracy and reaction times for all ‘same’ trials were recorded.

##### 2.1.3.3 Mental clock test

**Fig. A3:**
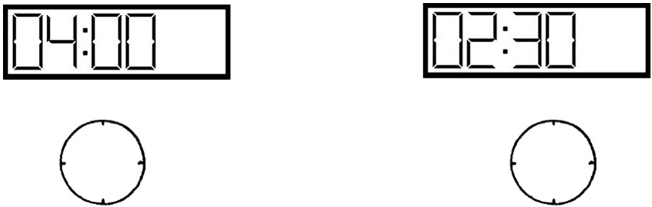
Example of an item from the Mental Clock Test (Paivio, 1978).

We used a computerized version of Paivio's (1978) clocks comparison task. In each trial, participants were presented with a pair of digital times, differing in the angle formed between the hour and the minute hands when they transformed on to an analog clock (i.e., 30°, 60°, 90°, 120°). Participants were asked to imagine two analog clocks showing these two times and choose the one creating a larger angle between the imagined clock hands (Fig. A3). Each of the four possible angular differences was presented in one of three locations on an imaginary analog clock: on the left half (of the analog clock), on the right half and in a left-right condition, yielding 12 conditions and 48 pairs of times.

A second list of 48 pairs of times was prepared by inverting the left-right locations of the pairs from the first list. Time pairs from both lists were mixed and presented in random order. There were 12 practice trials, followed by two blocks of 48 trials (96 trials in total). Each trial began with a fixation point “+” for 1000 ms prior to the onset of the task. The pairs of times were presented until a response was made. Response accuracy and reaction times were recorded.

##### 2.1.3.4 Object perspective test/perspective taking/spatial orientation test

**Fig. A4:**
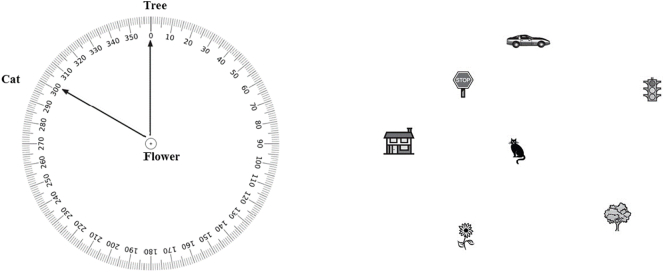
Example of an item from the Object Perspective Test/Perspective Taking/Spatial Orientation Test (Hegarty & Waller, 2004; Kozhevnikov & Hegarty, 2001).

We used a computerized version of the test developed by Kozhevnikov and Hegarty (2001), and revised by Hegarty and Waller (2004). In each trial, participants were shown a picture presenting configurations of seven different objects and an image of a full circle showing the imagined station point in the center of the circle and an arrow pointing to an imagined heading (Fig. 4, Fig. A4 below). Participants were instructed to imagine themselves standing at the location of one object (e.g., at the location of the flower in Fig. 4, Fig. A4) while facing another object (e.g., the tree in Fig. 4, Fig. A4), the latter becoming the heading at the 12th hour position. Then they were asked to indicate the angle formed with a third target object (e.g., the cat in Fig. 4, Fig. A4, where the correct answer should be 60°). Participants entered their answers by writing down the angle in degrees (indicating the direction of the target object). There were 12 items (and trials) in all. Participants were given one practice trial before the actual test. Each trial began with a fixation point “+” for 500 ms and was followed by a blank slide for 1000 ms. Then, the stimuli appeared and remained on the screen until the participant made a response. The score for each item was the absolute deviation in degrees between the correct directions and the participant's response. The total score was the average of all the deviations for all 12 items.

**Fig. 1:**
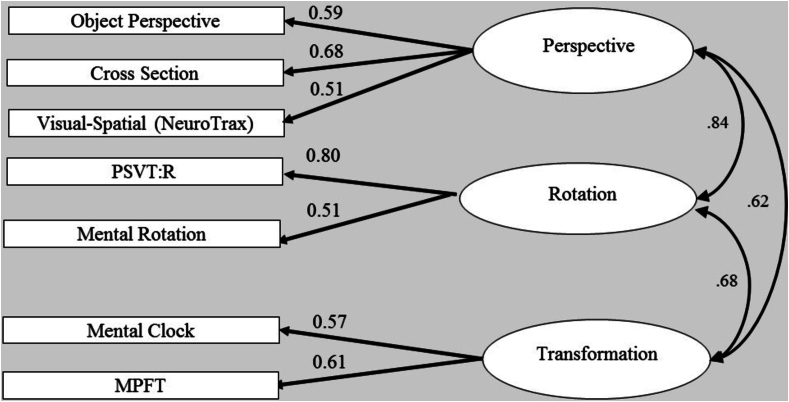
Confirmatory factor analysis - three-factor model. The ellipses represent the functions (latent variables), and the rectangles represent the tasks (measured variables) selected to tap the specific factor. The straight arrows represent loadings of specific tasks on the related factor. The curved double-headed arrows are correlations between the latent variables.

**Fig. 2:**
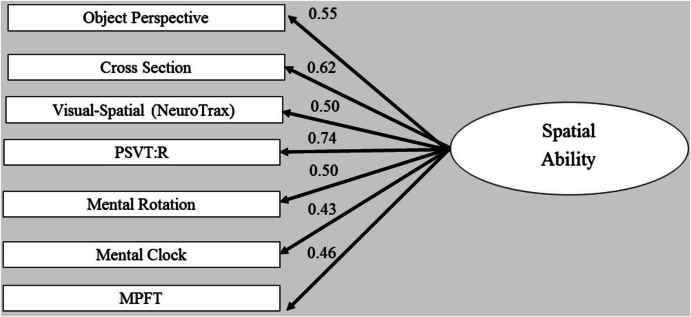
Confirmatory factor analysis - single-factor model. The ellipse represents the function (latent variable), and the rectangles represent the tasks (measured variables) selected to tap the specific factor. The straight arrows represent loadings of the specific task on the related factor.

**Fig. 3:**
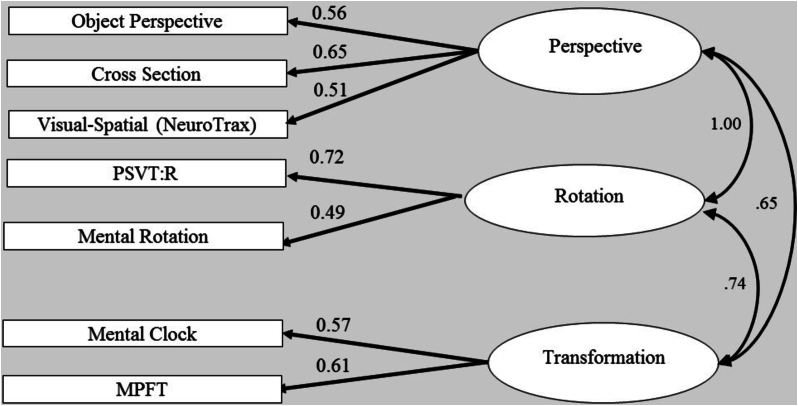
Confirmatory factor analysis nested - two-factor model. The ellipses represent the functions (latent variables), and the rectangles represent the tasks (measured variables) selected to tap the specific factor. The straight arrows represent loadings of specific tasks on the related factor. The curved double-headed arrow is the correlation. The correlation between perspective and rotation factors was set to 1.

**Fig. 4:**
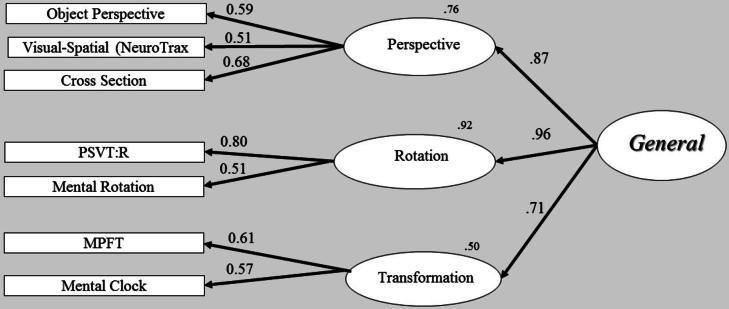
Structural model analysis - second-order factor model. One second-order factor (general) and three first-order factors (perspective, rotation and transformation). The straight arrows represent casual effect of the second-order factor on the first-order factors. Thus, each first-order factor has a residual error.

**Fig. 5:**
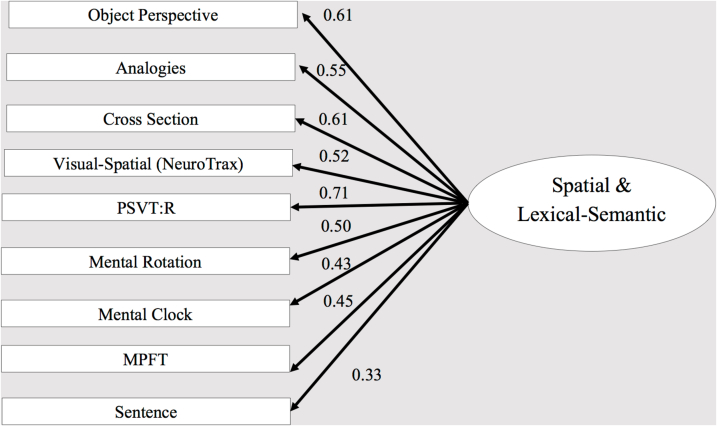
Confirmatory factor analysis - single-factor model. The ellipse represents the functions (latent variables), and the rectangles represent the tasks (measured variables) selected to tap the specific factor. The straight arrows represent loadings of the specific task on the related factor.

##### 2.1.3.5 Santa Barbara solids test/cross section test

We used a computerized version of the Cross Section Test (Cohen & Hegarty, 2012). Participants were shown an image of a geometric solid, crossed by a cutting plane. The test contained three levels of complexity of the geometric solids: simple, joined and embedded figs. (10 each), and two cutting plane orientations: orthogonal (horizontal or vertical) and oblique to the main vertical axis. Participants were required to choose from four options, the one showing correctly how the cut object would look after it was crossed by a cutting plane as indicated, when the cross section was looked at directly. The test consisted of 30 pictures (and trials) in all. Participants were given one practice trial before the actual test. Each trial began with a with a fixation point “+” for 500 ms prior to the onset of the task slide. The slide remained on the screen until the participants responded. Response accuracy and reaction times were recorded.

##### 2.1.3.6 Visual-spatial test (NeuroTrax Corp., Texas)

**Fig. 6:**
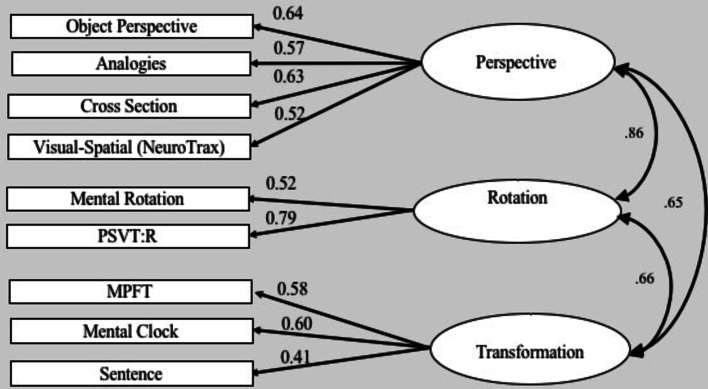
Confirmatory factor analysis - three-factor model (perspective, rotation and transformation). The ellipses represent the functions (latent variables), and the rectangles represent the tasks (measured variables) selected to tap the specific factor. The straight arrows represent loadings of specific tasks on the related factor. The curved double-headed arrows are correlations between the latent variables.

**Fig. A6:**
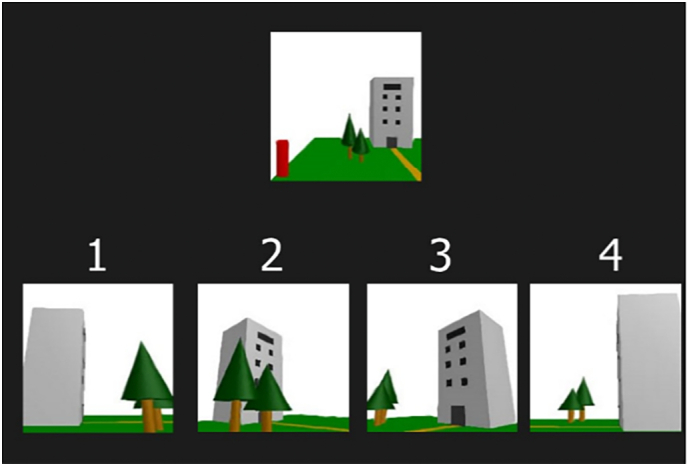
Example of an item from the Visual-Spatial Test (NeuroTrax Corp., Texas).

We used a computerized version of the visuospatial processing subtests from the NeuroTrax computerized neuropsychological battery. In each trial, participants were shown a slide presenting an image of an everyday scene with a red pillar in the top part (the target). Four images were given on the bottom part of the same slide, presenting four possible views of this scene as observed from different locations around it (Fig. 6, Fig. A6). Participants were instructed to imagine standing at the location of the red pillar and select one option out of four, showing the scene correctly as viewed from the location of the red pillar.

The test consisted of sixteen items and began with three practice trials, in order to familiarize the participants with the task requirement. Each trial began with a fixation point “+” for 1000 ms prior to the test itself. The slide remained on the screen until the participants responded. Response accuracy and reaction times were recorded.

##### 2.1.3.7 Mental paper folding test

**Fig. 7:**
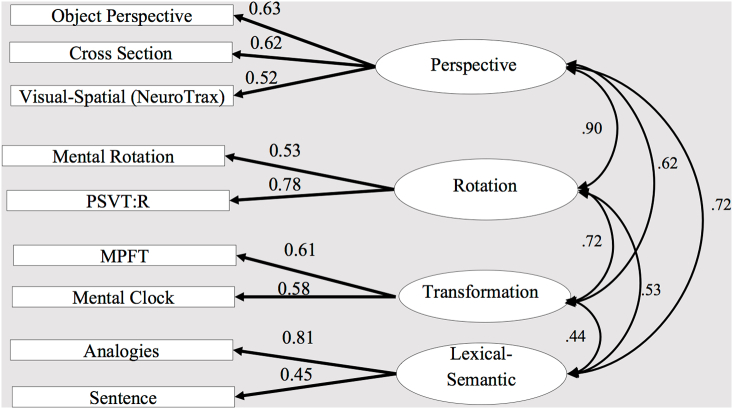
Confirmatory factor analysis - four first-order factors (perspective, rotation, transformation and lexical-semantic). The ellipses represent the functions (latent variables), and the rectangles represent the tasks (measured variables) selected to tap the specific factor. The straight arrows represent loadings of specific tasks on the related factor. The curved double-headed arrows are correlations between the latent variables.

**Fig. A7:**
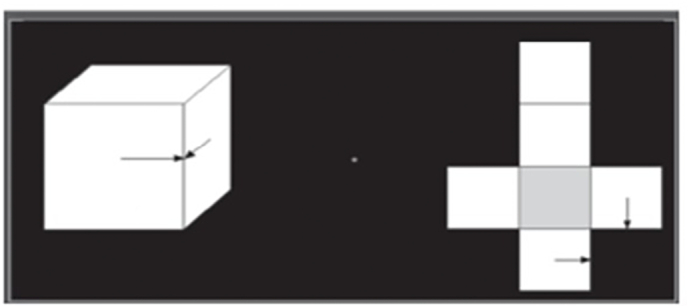
Example of an item from the Mental Paper Folding Test (Wright et al., 2008).

We used a computerized version of the Mental Paper Folding Test developed by Wright et al. (2008) (Fig. 7, Fig. A7). Participants were presented with an unfolded cube on the right side of the screen, and an image of a completed cube on the left side. One side of the completed cube and one side of the unfolded cube were shaded to mark the top or the base of the cube. Two arrows were drawn on each of the images. Participants were required to mentally fold the unfolded cube, and decide whether the two arrows on the cube would come together in the same way (‘same’) or not (‘different’). The items differed in difficulty, with the most complex items requiring 4–7 folds and rotations, and the less complex items requiring only one fold to provide the answer.

There were 12 practice trials, followed by 132 trials. Each trial began with a fixation point “+” for 500 ms prior to the test. The slide remained on the screen until the participants responded. Response accuracy and reaction times were recorded.

#### 2.1.4 Procedure

Participants were tested in groups of up to 12 students in one session, lasting approximately 1.5 to 2 h. Two sequences of the tests were generated and administered to two randomly chosen groups of participants. The seven tests were arranged in descending order of their respective lengths in one sequence, and task order was reversed in the second sequence. One list of tests was ordered as follows: Mental Paper Folding Test, Visual-Spatial Test (NeuroTrax), Santa Barbara Solids Test/Cross Section Test, Mental Rotation Test, Perspective Taking/Spatial Orientation Test, Mental Clock Test, and the Revised Purdue Spatial Visualization Test: Visualization of Rotations (Revised PSVT: R). The presentation order of the 2nd list was reversed.

#### 2.1.5 Scoring

The dependent measure for each task was the proportion of correct answers. Since we were mainly interested in the association and dissociation among the mental processes utilized in solving the different tasks (i.e., whether or not participants could succeed in solving an item) and less in the duration of achieving a correct solution, we did not use response time as a dependent variable. However, we did measure reaction time for a different purpose, as will be described below. Three tasks were scored as follows. Scores of the Perspective Taking Test were based on measuring the degrees of errors in estimating objects' positions. Thus, a higher score on this task indicated poorer performance. To simplify and keep all scores positive, we subtracted this average error score from 180°; thus, higher scores indicated better performance.

The scores used for two tasks—the Mental Rotation Test and the Mental Paper Folding Test—were taken for all participants only for those levels in which no floor or ceiling effects were observed, that is, to avoid using data for task levels that were found to be too easy or too difficult. Thus, these two of the tasks were characterized by differences in the levels of the complexity of items, due to the extent of the transformational and rotational degrees required (these levels of varying complexities were confirmed by the actual results). Some of the simple task levels (e.g., the 0° comparison of the Mental Rotation Test) could be solved effortlessly by most participants (cf., Lohman, 1986; Mumaw & Pellegrino, 1984). Accordingly, we selected for analyses only the proportion of correct answers in the medium levels, for these two tasks (we used the medium level and not the most difficult one in order to avoid a floor effect). Moreover, more difficult levels tend to associate with increased complexity, which might trigger the involvement of other cognitive constructs, such as working memory (Miyake et al., 2001; Mumaw & Pellegrino, 1984; Shah & Miyake, 1996). Thus, the Mental Rotation Test score was the average of the proportion of correct answers for 50° to 100° rotational angles. The Mental Paper Folding Test score was the proportion of correct answers for the easy and the medium levels (which required 1–4 mental folds).

In order to avoid any specific effect due to our selection of the aforementioned levels of difficulty, we used the mean of all difficulty levels for the Mental Rotation Test and the Mental Paper Folding Test.

Since including all levels of difficulty did not result in a noticeable change in factor loadings or in model fits, but negatively affected the normality requirement, we used the chosen difficulty^1^ levels, as described above, for these two tasks.

In the present study, we measured spatial ability by using computerized versions of known tests. In all of these measures, performance patterns were similar to patterns reported previously on paper-and-pencil tests. For example, reaction time on the Mental Rotation Test increased as a function of rotational angle, and was longer for 150° compared to 50°. Although we did not use reaction times as a dependent variable, collecting reaction times allowed us to confirm that the computerized versions of the tests were comparable to the paper-and-pencil versions. Moreover, response time and the known pattern of results could be used for unofficial motivational or effort screening—we would expect participants with low motivation to show a different, or unusual pattern of results (e.g., longer response times for the easy levels than for the difficult ones). We did not observe any unusual pattern in the current sample.

### 2.2 Results

Multivariate analysis is sensitive to outliers and assumes normal distribution. Therefore, data screening was used to restrain the influence of outliers on the results. For each participant, any score exceeding 2.5 standard deviations from the mean was fixed at a value equal to 2.5 standard deviations from the mean. This procedure affected five observations of the Mental Rotation and Perspective Taking Tests, three observations of the Paper Folding and Visual-Spatial (NeuroTrax) Tests, and two observations of the Mental Clock Test.

The dependent measure for each task was the proportion of correct answers. Descriptive statistics are given in Table 2. Note that for a few tests, skewness departed slightly from normality. However, Mardia's (1970) multivariate kurtosis was 3.97, satisfying the multivariate normality assumption. Reliability estimates (Cronbach α) for all measures demonstrated satisfactory internal reliability. Table 3 shows correlations among the spatial ability tests.

**Table 2:** Descriptive statistics for study variables.

Variable	Range	Mean	SD	Skewness	Kurtosis	Reliability
Perspective taking	149	0.48	41	−1.28	0.67	0.87
Mental rotation	0.69	0.82	0.16	−1.45	1.97	0.96
Cross section	0.90	0.48	0.21	−0.10	−0.73	0.87
PSVT: R	0.76	0.46	0.16	0.011	−0.47	0.76
Mental clock	0.38	0.88	0.088	−1.39	1.61	0.91
Mental paper folding	0.65	0.85	0.16	−1.04	0.08	0.93
Visual-spatial (NeuroTrax)	0.48	0.82	0.11	−0.61	0.04	0.45

Note. PSVT: R = the Revised Purdue Spatial Visualization Test: Visualization of Rotations.

**Table 3:** Correlations of the dependent measures.

Variable	1	2	3	4	5	6	7
1 Perspective taking	1						
2 Mental rotation	0.168	1					
3 Cross section	0.419^⁎⁎^	0.281^⁎⁎^	1				
4 PSVT: R	0.392^⁎⁎^	0.410^⁎⁎^	0.471^⁎⁎^	1			
5 Mental clock	0.257^⁎⁎^	0.222^⁎^	0.157	0.321^⁎⁎^	1		
6 Mental paper folding	0.232^⁎⁎^	0.229^⁎^	0.268^⁎⁎^	0.319^⁎⁎^	0.349^⁎⁎^	1	
7 Visual-spatial (NeuroTrax)	0.298^⁎⁎^	0.311^⁎⁎^	0.322^⁎⁎^	0.331^⁎⁎^	0.205^⁎^	0.207^⁎^	1

*p < .05, **p < .01.

CFA was performed using the AMOS program (Arbuckle, 1989), with maximum-likelihood estimation of the fitness measures. In general, several *goodness of fit* indicators are used to assess each model (Byrne, 2010; Hu & Bentler, 1999).

A three-factor model was tested to address the possibility of dissociation between the perspective taking factor, rotation, and transformation factors. The three-factor model is illustrated in Fig. 1 below, and its relevant fitness measures are reported in Table 4 (first line). The overall statistics for the three-factor model indicated an excellent fit, with non-significant χ2: χ2 (11) = 7.73, *p* = .73, χ2/df = 0.7. In addition, the SRMR was 0.03, the RMSEA was 0.00, and the CFI was 1.00. Furthermore, all the tasks significantly loaded on their presumed factors and the correlations among the three factors were significant and quite similar to the previous, two-factor model of Hegarty and Waller (2004) and of Kozhevnikov and Hegarty (2001). It should be pointed out that a simple model with a relatively small-to-medium sample size must achieve stricter cutoff values for an acceptable model fit (Hair et al., 2009; Kenny et al., 2015). Thus, the three-factor model seems to have achieved these strict criteria and provided an excellent fit to the data.

**Table 4:** Fit Index for the Three-Factor Model using Confirmatory Factor Analysis.

Model	χ2	df	χ2/df	RMSEA	SRMR	CFI	NFI
1. Three-Factor model	7.73	11	0.70	0.00	0.03	1.00	0.95
2. Single-Factor model	16.31	14	1.16	0.03	0.05	0.98	0.90

Two-Factor models							
3. Perspective = Rotation	10.25	12	0.85	0.00	0.04	1.00	0.94
4. Perspective = Transformation	14.67	12	1.22	0.04	0.06	0.98	0.91
5. Rotation = Transformation	12.63	12	1.05	0.02	0.04	0.99	0.92

Structural model							
6. Second-Order model	9.26	12	0.70	0.00	0.03	1.00	0.94

Note. No Chi-square values were significant. RMSEA = root mean square error of approximation; SRMR = standardized root mean square residual; CFI = comparative fit index; NFI = normed fit index.

This model was also confirmed by performing statistical comparisons of the proposed three-factor model with different models. Thus, we first examined a comparison to a single-factor model, exploring the possibility that all tasks were driven by a single factor (see Fig. 2). We compared the three-factor model to a single factor, by constraining all the correlations between the three factors to be 1, so that the single model was nested within the three-factor model. The overall measures for this model provided a reasonable fit to the data, as summarized in Table 4 (second line). However, the χ2 test of the difference between these two models produced a significant result, χ2 (3) = 8.58, *p* = .03, indicating that the three-factor model fit the data significantly better than the single-factor model. Therefore, a second step was undertaken, namely, comparing a two-factor model fit to that of the three-factor model. Specifically, we compared between an alternative two-factor model, nested within the three-factor model, by constraining the correlations between each two factors (of the three-factor model) to 1, with the other two of the remaining correlations between factors left ‘free’ to be estimated (i.e., not constrained to a specific value). Although all of the alternative two-factor models (models 3–5 in Table 4) provided an overall acceptable fit to the data of a two-factor model, the χ2 tests of the differences indicated a significantly better fit of the three-factor model over the two-factor model (Perspective = Transformation, χ2 (1) = 6.94, *p* = .01; Rotation = Transformation, χ2 (1) = 4.90, *p* = .03). Note that in the first two-factor model (third data line in Table 4, Fig. 3), in which we set the correlation between perspective taking and rotation factors to be 1, the χ2 test of the difference was not significant at the 0.05 level, indicating that this model provides as good a fit to the data as the three-factor model does (Perspective = Rotation, χ2 (1) = 2.51, *p* = .11). However, all the other χ2 difference tests indicated a significantly better fit of the three-factor model over the two-factor models. The result of the first two-factor model (third line in Table 4) suggests that tasks requiring a rotational component share substantial variance with tasks in the perspective domain. This finding is also in line with previous finding. A possible explanation offered was the shared variance among rotation and perspective tests. Accordingly, Kozhevnikov and Hegarty (2001) found that most participants reported using an egocentric strategy to solve the Object Perspective Taking Test, yet not all of them, as some of the participants reported using an object-rotation strategy to solve this test (Boer, 1991). Finally, this finding may be explained by the proposal, mentioned in the Introduction section regarding spatial tests, that a common, single process may be shared by these spatial factors.

#### 2.2.1 Second-order analysis

Although the proposed three-factor model exhibited a better fit compared with the other two-factor models, the three components are correlated. Therefore, it is possible that many of the tests, assumed to reflect different factors, may share some operations (i.e., encoding, maintaining), which could explain the respectable correlations among the three factors in the current study and between perspective taking and rotation factors in previous studies (Hegarty & Waller, 2004; Kozhevnikov & Hegarty, 2001). Pursuing this idea further, it is conceivable that the correlations among these three visual-spatial factors may be explained by a single higher-order factor, which represents a more general process compared with the first-order factors that reflect specific visual-spatial processes. That is, when a second-order factor is hypothesized to explain the pattern of relations among first-order factors, the higher-factor captures some common underlying process of the first layers of factors. As such, the second-order factor reflects an overarching construct. Accordingly, the structural model^2^ is a more parsimonious explanation for the correlations among the first-order factors, compared to the measurement model (Brown, 2015; Byrne, 2010; Hair et al., 2009; Keith, 2014).

Statistically, a three-factor model is equivalent (the same degrees of freedom and model fit) to a second-order model with three first-order factors. Thus, a second-order model with three first-order factors would provide a ‘just-identified’ solution and the two models would fit equally well statistically.^3^

The higher second-order model is illustrated in Fig. 4 and its relevant fitness measures are reported in Table 4 (model 6). Note that the fit and the first-order loadings on the measures are identical to those of the three-factor model, as this structural model is just- identified, with the addition of the paths between second- and first-order factors. We used the common solution for just-identified models and constrained the residual variances of two first-order factors that showed nonsignificant differences (critical ratio is <1.96): rotation and perspective taking. The degrees of freedom for this model increased from 11 to 12. The χ2 difference test was nonsignificant (χ2 (1) = 1.53, *p* = .22), indicating that this ‘constrained’ second-order model provided a good fit to the data as the ‘non-constrained’ second-order model (which is identical to the three-factor model). However, a comparison between the two models was not our main goal in this case, since we were interested in the paths between the first- and second-order factors. As can be seen in Fig. 4, Fig. A4, rotation and perspective taking, two highly correlated first-order factors, also loaded strongly and similarly onto the second-order factor (0.87–0.96). The similar loadings of these factors (rotation and perspective taking) onto the second-order factor raise a question regarding the distinctiveness of these factors. Since the second-order factor captures the common underlying operations of the three first-order factors, it may be that the strategies comprising these two factors (rotation and perspective taking), although assumed to reflect different factors, still share non-trivial features. Moreover, it may be that these supposedly ‘pure’ visual-spatial factors contain also non-spatial properties, which cannot be detected by using only visual-spatial measures. In the following study, we address this possibility by adding non-spatial tests.

### 2.3 Discussion and conclusions

The goal of the first study was to elucidate the nature of spatial skills by exploring the underlying visual-spatial components and the relations among them. In this study, we were inspired by a recent proposed dual-factor model of Kozhevnikov and Hegarty (2001) and Hegarty and Waller (2004), but propose the addition of another factor we called transformation. We selected a group of tasks commonly used in the field of cognitive visual-spatial research, with the aim of representing closely the range of these abilities. The results of the CFA support a tripartite model of spatial abilities. A closer look at the tasks for each factor seems to motivate a possible explanation regarding the relationship among these three factors, reflected in the correlations among them. It appears that these factors reflect an essential stage consisting of visual-spatial strategies taking place during different steps of spatial problem solving. We also found significant and relatively high correlations among the three factors, suggesting they may share a common underlying process. The high and consistent correlations between first-order factors present some difficulty in discriminating between factors. To address this issue, we examined whether a second-order factor could explain the correlations among the first-order factors.

The structural analysis of the second-order model showed a good fit to the data, similar to the initial three-factor model, accounting well for the correlations among the three first-order factors. Second-order structural model analysis provides additional support for the three-factor model, indicating some degree of a common, underlying cognitive process for the three components of rotation, perspective, and transformation. We regard this underlying factor as reflecting mental manipulations. In what follows, we will explore the possibility that the second-order model reflects an overarching process and is not restricted to spatial ability.

## 3 Study 2

Within the present paper, we suggested that the structural model, which was presented in the first section, represents overarching processes and is not limited to the spatial domain. Thus, it is possible that the first-order or sub-factors (rotation, perspective, and transformation) are involved in different types of performance and settings, even beyond visual-spatial skills, as we hypothesized that underlying all cognitive skills there is a common factor we referred to as mental manipulation. In the current study, we examine this suggestion by using both visual-spatial and lexical-semantic measures. Specifically, we invited the participants from the first stage for a second testing session, which included two lexical-sematic tests. Thus, the current analysis is based on the seven visual-spatial tasks from the previous study, and two lexical-semantic tests: verbal analogies and sentence completion. These two verbal tasks may be useful for measuring general semantic ability in that they require participants to identify relational patterns (e.g., identify the relationship between a pair of words in the verbal analogy test). These tests are hypothesized to tap an underlying mental-analytic process, which could be parallel to the process involved in performance on spatial tests. Thus, it is hypothesized the verbal analogy test would load on the perspective-taking factor as both tests require abstracting a mental entity from the concrete stimulus and then manipulating it (acting on it as the task requires); in other words, operating on abstracted representations (e.g., situations, objects, shapes, ideas) from the concrete items. The verbal analogy test requires classifying the relationship between a criterion pair of words and matching it with an abstracted relationship of another pair, which resembles it most closely. Gentner (1983) described the mapping component, which emphasizes the process of identifying commonality between the target and the corresponding if there is a common underlying factor that is a domain-general, pair. For example, consider the verbal analogy test. It requires reviewing word meanings, so that the two words of the pair join mentally into the same concept (e.g., hand and flag – you wave both to signal some meaning). By a similar argument, it is suggested that a sentence completion test should load on the transformation factor: both require mental matching of the parts before achieving a solution. For example, the sentence completion task requires the construction of an optional model of the abstract meaning before binding the different parts, so that a coherent sentence is formed. Similarly, the MPFT and the Mental Clock Test require the construction of an optional whole (a cube in one test, clock faces in the other) before matching parts to arrive at the solution.

Adding measures from a non-spatial domain could produce a new latent variable (by loading on a verbal factor) or load on the same latent variables that represent spatial operations. It follows reasonably from the foregoing that lexical-semantic tasks might be better accounted for within the 3-factor model above, and not by a fourth, purely verbal factor, as these tasks arguably share a degree of commonality with perspective-taking and transformation factors. Moreover, assuming that the non-spatial tests load on the same (spatial) components would support the idea of a higher-order factor, not specific to spatial processing.

This hypothesis was put to the test by considering two main structural models. The first model presents the presumably traditional view that the verbal tasks should constitute a four-factors model. This structural model presents a single second-order factor with four first-order factors: a new lexical-semantic factor and the three from the first study. In this model, the two lexical-semantic measures would produce a new latent variable, which reflects a specific lexical-semantic factor. The second structural model presents the possibility that the three-known visual-spatial factors can be explained by a higher second-order overarching factor, as in the first study above. In this structural model, the two lexical-semantic tests are expected to load on the existing three factors: rotation, perspective, and transformation. Specifically, we examine whether a single second-order factor with the three first-factors (perspective taking, rotation and transformation) model would fit the data after adding the two lexical-semantic tests.

### 3.1 Method

#### 3.1.1 Participants

Out of the original 133 participants in Study 1, 128 undergraduate students returned for the second session, which was held after a period of a month to two months following the first session.

#### 3.1.2 Materials

The participants completed two computerized semantic tests, which were programmed using *E*-prime version 2.0.10.356 software (Psychology Software Tools, Pittsburgh, PA). The items for the two lexical-semantic tests were selected from the verbal subtest of the Psychometric Entrance Test (PET), a test used for selective admission to universities in Israel (used with permission of the National Institute for Testing and Evaluation, Israel). The tests are similar to the Verbal Scholastic Aptitude Test (SAT).

##### 3.1.2.1 Verbal analogies test

Participants were presented a criterion pair of words and were instructed to identify the relationship between them. Participants were required to choose from four word pairs, appearing on the same slide, the one pair resembling most closely the relationship between the criterion pair (see below). The test consisted of 20 multiple-choice analogies. Each trial began with a fixation point “+” for 500 ms and was followed by a target slide that remained on the screen for 4 s. Then a blank slide appeared for 750 ms. Accuracy and reaction times were recorded.

An example of an item from the Verbal Analogy Test (PET, National Institute for Testing and Evaluation, Israel):

unstable: collapsed -

(1) adjacent: moved away
(2) clear: was clarified
(3) fractured: healed
(4) flammable: caught fire

##### 3.1.2.2 Sentence completion test

In each trial, participants were shown a slide presenting a sentence with one or more missing sections, followed by four possible words or statements for completion of the sentence. Participants were instructed to choose the one option that best completed the sentence, so its content was coherent (see below). The test consisted of 12 multiple-choice problems. Each trial began with a fixation point “+” for 500 ms and was followed by a target slide that remained on the screen for 4 s. Then a blank slide appeared for 1000 ms. Accuracy and reaction times were recorded.

An example of an item from the Sentence Completion Test (PET, National Institute for Testing and Evaluation, Israel):

Despite the ___the new calculator, employees were afraid to demand that use of it be ___, because it was developed by Zorkin. Now that Zorkin has ___ the respect of management, it seems ___.

(1) many problems caused by/discontinued/lost/highly improbable that they will make this demand
(2) advantages of/expanded/gained/they will refrain from making this demand
(3) many problems caused by/discontinued/gained/they will insist on this demand
(4) advantages of/expanded/gained/their fear will vanish and they will make this demand

## 4 Results

The dependent measure for each task was the proportion of correct answers; thus, higher scores indicated better performance. Descriptive statistics are given in Table 5. Table 6 shows correlations among all the tests (Study 1 & 2). As already mentioned above, adding measures from a non-spatial domain could produce a new latent variable (by loading on a lexical-semantic factor) or load on the same latent variables that represent visual-spatial ability (Study 1). These two possibilities were tested by first considering two measurement models, with three and four factors, as illustrated in Fig. 6, Fig. 7, respectively. Their fitness statistics are reported in Table 7. The overall statistics for these two models indicated a good fit to the data, with all tests significantly loaded on their presumed factor. In the first stage, comparison of the two models to a single-factor model was performed, assuming that all tasks were driven by a single spatial factor (Fig. 5). All the fit indices of the two structural models provided a better fit to the data than the single-factor model did. This was confirmed by comparison of the models with a χ2 difference test, indicating a significantly better fit of the two measurement models (Table 7) than the single-factor model, (χ2 (3) = 11.53, *p* < .01; χ2 (6) = 18.32, *p* = .00, respectively). The second stage was comparing the two models to each other, as each model represents a different theoretical framework. The two measurement models provided a suitable fit to the data: the χ2 tests of the difference was not significant at the 0.05 level, indicating that the four-factor model did not provide a better fit to the data than the three-factor model (χ2 (3) = 6.79, *p* = .07).

**Table 5:** Descriptive statistics for study variables.

Variable	Range	Mean	SD	Skewness	Kurtosis	Reliability
1 Perspective taking	149	0.48	41	−1.28	0.67	0.87
2 Mental rotation	0.69	0.82	0.16	−1.45	1.97	0.96
3 Cross section	0.90	0.48	0.21	−0.10	−0.73	0.87
4 PSVT: R	0.76	0.46	0.16	0.011	−0.47	0.76
5 Mental clock	0.38	0.88	0.09	−1.39	1.61	0.91
6 Mental paper folding	0.65	0.85	0.16	−1.04	0.08	0.93
7 Visual-spatial (NeuroTrax)	0.48	0.82	0.11	−0.61	0.04	0.45
8 Verbal analogies	0.60	0.70	0.14	−0.45	−0.24	0.58
9 Sentence completion	0.80	0.68	0.18	−0.32	−0.56	0.60

Note. PSVT: R = the Revised Purdue Spatial Visualization Test: Visualization of Rotations.

**Table 6:** Correlations of the dependent measures.

Variable	1	2	3	4	5	6	7	8	9
1 Perspective taking	1								
2 Mental rotation	0.168	1							
3 Cross section	0.419^⁎⁎^	0.281^⁎⁎^	1						
4 PSVT: R	0.392^⁎⁎^	0.410^⁎⁎^	0.471^⁎⁎^	1					
5 Mental clock	0.257^⁎⁎^	0.222^⁎^	0.157	0.321^⁎⁎^	1				
6 Mental paper folding	0.232^⁎⁎^	0.229^⁎^	0.268^⁎⁎^	0.319^⁎⁎^	0.349^⁎⁎^	1			
7 Visual-spatial (NeuroTrax)	0.298^⁎⁎^	0.311^⁎⁎^	0.322^⁎⁎^	0.331^⁎⁎^	0.205^⁎^	0.207^⁎^	1		
8 Verbal analogies	0.438^⁎⁎^	0.168	0.289^⁎⁎^	0.374^⁎⁎^	0.176^⁎^	0.187^⁎^	0.298^⁎⁎^	1	
9 Sentence completion	0.236^⁎⁎^	0.071	0.096	0.134	0.277^⁎⁎^	0.205^⁎^	0.211^⁎^	0.366**	1

*p < .05, **p < .01.

**Table 7:** Fit index for the three-factor model using confirmatory factor analysis.

Model	χ2	df	χ2/df	RMSEA	SRMR	CFI	NFI
Measurement model							
1. Single-factor model	43.21	27	1.60	0.07	0.06	0.92	0.82
2. Three first-order-factors	31.68	24	1.32	0.05	0.05	0.96	0.87
3. Four first-order-factors	24.89	21	1.18	0.04	0.05	0.98	0.89

Structural models							
4. Three first-order-factors with constrained residual	32.42	25	1.30	0.05	0.05	0.96	0.86
5. Four first-order-factors	27.96	23	1.22	0.04	0.05	0.99	0.88

Note. Chi-square values were significant only for the first model (single-factor model). RMSEA = root mean square error of approximation; SRMR = standardized root mean square residual; CFI = comparative fit index; NFI = normed fit index.

Although both measurement models were considered to fit the data, they still differed in the correlations among the factors. Specifically, in the four-factor model (Fig. 7), the high correlations between perspective taking and rotation factors (*r* = 0.90), and the correlations between these two factors and the new lexical-semantic factor (*r* = 0.72), make it difficult to distinguish between the factors. Moreover, since a four-factor model actually splits between the spatial factors and verbal factor, we would have expected this model to show a significantly better fit than a model that combines these two domains, if indeed the verbal tasks constitute a distinct mental factor, as is traditionally conceptualized (cf. Fodor's (1983) The Modularity of Mind). Evidently, this was not the case; the three-factor model was found to well suit the data and supported the suggestion of an overarching, possibly even unifying, ability. Perhaps for that reason, the two lexical-semantic tests in the three-factor model (Fig. 6) were significantly loaded on two assumed-to-be ‘spatial’ factors (perspective taking and transformation), with the correlations among the three factors being moderate and very similar to the finding in Study 1. It appears that the present findings support the possibility that the three-factor model represents broader underling cognitive processes.

**Fig. 8:**
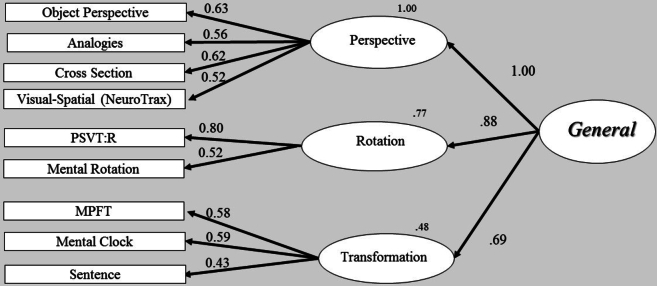
Structural model analysis: second-order factor model. A single second-order factor (general) and three first-order factors (perspective, rotation, transformation). The straight arrows represent causal effect of the second-order factor on the first-order factors. Thus, all first-order factors have residual errors. Residual variances of two first-order factors, rotation and perspective were constrained to be equal.

**Fig. 9:**
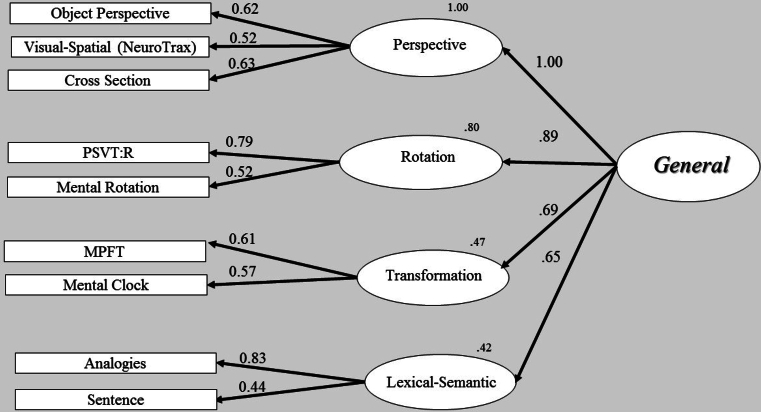
Structural model analysis: second-order factor model. A single second-order factor (general) and four first-order factors (perspective, rotation, transformation and lexical-semantic). The straight arrows represent causal effect of the second-order factor on the first-order factors. Thus, all first-order factors have residual errors.

In order to determine whether the second-order models would fit the data, we tested two structural, second-order models. The first model was a second-order factor with three first-order factors. Note that in this first second-order model (Fig. 8), we used a common solution for a ‘just-identified’ model and constrained the residual variances of two first-order factors (rotation and perspective taking) to be equal. In short, this solution increases degrees of freedom and allows us to test a second-order model with three first-order factors (Brown, 2015; Byrne, 2010; Hair et al., 2009; Keith, 2014). The second structural model also contained a second-order factor but with four first-order factors; a new lexical-semantic (verbal) factor and the three spatial factors. The two models are illustrated in Fig. 8, Fig. 9 respectively, and their fitness statistics are reported in Table 7. The overall statistics for these two higher-order models indicated a good fit to the data as summarized in Table 7, with all the first-order factors having significantly loaded on their second-order factor. We compared the structural models to a single-factor model (first line in Table 7). A single-factor model assumes that all tasks are driven by a single factor. It is interesting to note that all the fit indices of the structural models provided a better fit to the data than the single-factor model did. This was also confirmed by comparing the models with a χ2 difference test, indicating a significantly better fit of the second-order factor models over the single-factor model, (χ2 (2) = 10.79, *p* = .00; χ2 (4) = 15.25, *p* = .00, respectively).

The next step was to assess the structural models, since each model represented a different theoretical approach. These two structural models provided a suitable fit to the data; the χ2 test of the difference was not significant at the 0.05 level (χ2 (2) = 4.46, *p* = .10), indicating that the model with four first-factors (Fig. 9; perspective taking, rotation, transformation and lexical-semantic; i.e., the ‘verbal model’) did not provide a better fit to the data than the model with three first-order factors (Fig. 8; perspective taking, rotation and transformation; i.e., the ‘spatial model’). A closer look at the causal paths from the first-order factors to the second-order factor reveals a similar pattern of relations in both models. Of interest is that the second-order model with the three first-factors (Fig. 8), and the model with the four first-factors (Fig. 9) showed similar and significant loading of the first-order factors on the second order factor. As shown in Fig. 8, Fig. 9, the paths between the first- and second-order factors are very similar, with rotation and perspective taking, the two highly correlated first-order factors (see Study 1) also having loaded strongly on the second-order factor. The Transformation factor also provided similar loading on the second-order factor in these two models (*r* = 0.65 and *r = 0*.71, respectively). Note that the sentence completion test, a lexical-semantic task, significantly loaded on the Transformation factor, which is in line with our definition of this factor. The high loadings of first-order factors on the second-order factor (Fig. 8, Fig. 9) in both structural models indicate strong relations among first-order factors on the second-order factors, implying that the second-order model represents the data well. Note that although the perspective taking, first-order factor, provided identical and high loading for both structural models, this factor was not identical in the two models, and was constructed by different sets of tests: In the structural model with three first-order factors (Fig. 8), the perspective taking factor comprised three spatial tests and a single lexical-semantic test (i.e., the verbal analogy test). In contrast, in the structural model with four first-order factors (Fig. 9), this factor was formed by only three spatial tests, whereas the two lexical-semantic tests were nested in a different ‘verbal factor’. Splitting between the spatial and verbal domains, so each factor reflected more uniform processes, did not yield a better model than the model that integrated tests from spatial and verbal domains. Moreover, the structural model with three first-order factors (Fig. 8), in which the two lexical-semantic tests were significantly loaded on two presumed ‘spatial’ factors (perspective taking and transformation), is considered a more parsimonious model and supports the idea of these three first-order factors as representing overarching processes, cutting across spatial and verbal domains.

### 4.1 Discussion and conclusions

In Study 2, we attempted to examine our suggestion that a higher-order factor, which is domain-general in nature, can explain the pattern of correlations among the visual-spatial first-order factors. In the current preliminary study, we added two lexical-semantic tests to the visual-spatial tests of the first study. The results of the CFA and SEM indicated that the measurement and structural models fit the data better than a single-factor model. However, the measurement and structural models with four first-order factors (Fig. 7, Fig. 9) did not provide a better fit to the data than the models with three first-order factors (Fig. 6, Fig. 8). The structural model with three first-order factors, combining spatial and lexical-semantic tests, supports our hypothesis regarding the overarching nature of the second-order and the first-order factors, representing processes which cut across tests of different domains. Since this structural model (combining spatial and lexical-semantic tests) is also considered to be a more parsimonious model, we prefer this model over the extended four-factor model. These findings are also in line with our results (Study 1), of three well-differentiated, yet related, components of rotation, perspective and transformation.

### 4.2 General discussion

The goal of the current study was to elucidate the nature of the underlying components of visual-spatial processes and to examine the existence of a domain-general process beyond spatial ability. Specifically, in Study 1, another factor—transformation—was suggested to denote a basic visual-spatial process, involving the ability to perform changes, modifications, and re-combinations of concrete and abstract entities in our mind. Visual-spatial abilities have always been integral elements of intelligence. As such, it is possible that latent factors, considered the building blocks of visual-spatial skills, reflect overarching processes, namely mental manipulations, and are involved in other domains, for example, in lexical-semantic processing. To address this possibility, a structural model analysis was conducted in Study 2.

The results of the CFA support a tripartite model of spatial abilities. We labeled these three underlying factors as transformation, perspective-taking and rotation factors (presented in Fig. 1). Given the high correlations among visual-spatial factors in previous studies, we expected to find correlations of this factor (transformation) with the two other latent variables. Indeed, the transformation factor significantly correlated with the rotation (*r* = 0.68) and perspective-taking (*r* = 0.62) factors. We also found a significantly high correlation between rotation and perspective factors (*r* = 0.84), suggesting they may share a common process. The correlation between the domains of rotation and perspective-taking is consistent with previous findings (Hegarty & Waller, 2004; Kozhevnikov & Hegarty, 2001). Thus, performance on perspective-taking tasks is likely to be similar to the performance on rotation tasks, as both tasks seem to share a degree of adjustment of either objects or the observer's egocentric point of view, reflected in the high correlation between them. The relatedness of rotation to perspective taking (*r* = 0.84) is consistent with a similar, earlier suggestion by Lohman (1979). Lohman concluded that tasks dealing with orientation factors are often solved by rotation strategies, thus making these two factors share some mental operations during performance.

Our initial findings indicated that the three-factor model provided a significantly better fit to the data as compared with the single-factor model and most of the two-factor models.

However, relatively high correlations among these first-order factors present difficulty in discriminating between factors, and they may also indicate a common underlying higher factor. To address this problem, a structural analysis was performed to examine whether a second-order model could represent the data better. Hence, we hypothesized a second-order factor to account for the correlations among the first-order factors. To this end, a second-order model analysis was used. The result of the structural analysis provided a good fit to the data, with a good account for the correlations among the three first-order factors (with rotation and perspective taking first-order factors loading strongly on the second-order factor *r* = 0.87 and 0.96, respectively).

The possibility of an overarching process underlying not just visual-spatial processing but also lexical-semantic tasks, which provided the motivation for Study 2. In the current study, we added two lexical-semantic tests to the visual-spatial tests of the first study to test this hypothesis. Two structural models were our main interest in this second study: the first model reflected the assumption that a single second-order factor with three first-order factors (perspective taking, rotation and transformation, ‘spatial model’) would fit the data, even after adding two lexical-semantic tests. In the alternative model, the traditional dissociation between visual-spatial and lexical-semantic tests was examined, through a structural second-order model with four first-order factors: the three spatial factors and a fourth lexical-semantic factor. The high loadings of first-order factors on the second-order factor in both structural models indicated that the second-order models represented the data well. However, adding a separate verbal factor did not yield a better model than the model that integrated tests from the spatial and verbal domains. Our initial findings indicated that the four-factor model did not provide a significantly better fit to the data as compared with the three-factor model. The structural model with three first-order factors, combining spatial and lexical-semantic tests, is a more parsimonious model, and it follows that this model represents the data better than the extended structural four-factor model.

These findings of the measurements and results of the structural three-factor models support the overarching nature of the second-order factor. They also support our proposal regarding the general nature of the first-order factors, captured by tests of different domains (i.e., visual-spatial and lexical-semantic) and not only by ‘pure’ or specific spatial process. Thus, a ‘specific’ factor seems to capture just a limited or a relatively narrow range of the described ability. However, and as already suggested by Gerbing et al. (1994) and by Koufteros et al. (2009), these specific factors can be used as the building blocks of a second-order factor. According to Koufteros et al. (2009), “Each facet is conceptualized as one of the building blocks of the higher-order construct. The idiosyncrasy of each content domain is retained, while unidimensionality and discriminant validity can be addressed by the higher-order specification. ‘Bloated specific’ factors can be included as some or all of the constituent building blocks of higher-order structures, and highly correlated factors can now be related through what they all share, a common higher-order factor” (p. 641). Considering this view of specific and non-specific factors could explain the high correlation between perspective-taking and rotation factors, and the high loading of these factors on the second-order factor.

To summarize, both structural models fit the data well. SEM results indicated that the best-fitting model is a structural model with only three first-order factors (Fig. 8), combining visual-spatial and lexical-semantic tests. This model represents a more parsimonious explanation for the pattern of the relation of the first-order factors and appears to reflect the multidimensional nature of the higher-order factor (Koufteros et al., 2009). In support of this general model, Vergauwe et al. (2010) found a trade-off between verbal and spatial processes in a dual-task condition. The authors proposed that verbal and visual-spatial processes rely on a shared domain-general component. This domain-general factor we referred to as mental manipulation, to capture not only its underlying role in human cognitive skills, but also its biological continuity across various species.

The present study is not without some limitations. Our study focused on components of spatial ability, without attending sufficiently to differences arising from other cognitive abilities (e.g., executive function and working memory), and correlations of these abilities with spatial skills. For example, individual differences and classification of latent variables were based on the level of performance (accuracy), without examining the specific strategy usage in each test. However, strategy usage studies are typically based on participants' self-reporting, which has its own limitations since the accuracy of self-reporting has been questioned (Lohman, 1979).

Another concern is the number of tests used to represent each latent variable. In some of the proposed models, rotation and transformation factors have two indicators each. However, we selected well-known and frequently used measures. Given the pattern of results among all the presented models (Studies 1 and 2), it appears that the chosen measures (spatial and lexical-semantic tests) adequately and closely represented the range of the theorized underlying skills. Although the reliability of the Visual-Spatial Test (NeuroTrax) was moderate, we decided against omitting it, as solving it seems to require an obvious perspective-taking process. It should be noted that even a robust model, containing more than two indicators for each of the latent factors, cannot ensure that the measures capture the full range of a skill. Nevertheless, further studies examining the proposed components and models with more indicators are desirable to confirm the present results.

Despite the modest sample size of the present studies, the proposed CFA and SEM models demonstrated very good fits to the data across different fit indexes, and provided a better fit than other alternative models. The structural models also offer supporting evidence for the first-order factors serving as building blocks of a higher-order latent variable. Nevertheless, the present results should be replicated using a larger sample. Finally, it is acknowledged that our model is based on a sample of college students, which may limit its generalizability across different populations (i.e., clinical patients, children, etc.).

## 5 Conclusion

The current paper is an attempt to sharpen our understanding of the underlying processes of visual-spatial aptitude and examine the existence of a more general overarching process, reflected in the visual-spatial domain and in lexical-semantic processes (and possible in other domains as well). The underlying second-order ‘general’ factor found in this study (perhaps representing intelligence), which is common to visual-spatial and lexical-semantic tasks, reflects what we referred to as mental manipulations (MM). We propose that intelligence is a collection of concrete and abstract mental capacities crucial for survival among humans and non-humans. These mental operations form a biologically based concept that evolved continuously from object manipulation to mental manipulation in animals and humans. We suggest that the operations we refer to as essential to intelligence (e.g., abstract transformations, rotations, perspective-taking, lexical-semantic tasks) are fundamentally acts of mental manipulations, which cut across domains. The present study offers new data to support such an approach to intelligence (See also Bar-Hen-Schweiger & Henik, 2020; Bar-Hen-Schweiger et al., 2017; Bar-Hen-Schweiger & Henik, 2019; Henik et al., 2021; Burkart et al., 2016; Sternberg, 2017, Sternberg, 2019). In these previous studies, the concept of an underlying basis of intelligent behavior, continuous across species, was developed. Here, we propose that the three components (transformation, perspective, and rotation) could reflect a building block comprising human intelligence, characterized by the ability to perform mental manipulations. According to our proposal, as was clarified in the current paper and other articles by our group, MM is the essence of intelligence.

In the first stage, we selected a group of tasks frequently used in the field of cognitive visual-spatial research, with the aim of representing closely the range of these skills. In order to improve prior models, a third factor—transformation—was added to perspective-taking and rotation factors. Thus, our data were found to be modeled best by three underlying factors, with an overarching higher-order factor we described as reflecting mental manipulation. We then performed a second study, adding lexical tasks, with the aim of examining whether there is a latent variable manifesting as an overarching concept. The results of CFA and SEM analyses indicated that the best-fitting, parsimonious model was the one with a single second-order factor, with three first-order factors combining visual-spatial and verbal tests. These first and second-order factors are presumed to reflect overarching processes or mental manipulations, in that they are suggested to be fundamental to a wide variety of mental activities, and are not restricted to the visual-spatial domain. That is, the SEM analyses indicated the existence of an underlying factor, which we termed mental manipulation, to underscore its continuity in various animal species, and its central factor in the human development of cognitive skills, such as visual-spatial and lexical-semantic abilities. In addition, the transition of object manipulation skills with other cognitive processes may be fertile ground for cross-disciplinary research efforts in several fields, such as neuroscience, neuropsychology, biology, and sociology, with the goal of developing neuroanatomically based measurements of such skills. As such, they may be implicated in broader aspects of human intelligent behavior. Naturally, our formulation here, albeit promising, is a first step. It requires replications and further validation by additional research, testing its modeling efficiency with larger samples and with a variety of additional tasks in additional domains.

## CRediT authorship contribution statement

**Moran Bar-Hen-Schweiger:** Writing – original draft, Methodology, Data curation. **Avishai Henik:** Writing – review & editing, Supervision.

## Ethical approval

The authors do not have any interest that might be interpreted as influencing the research and APA ethical standards were followed in the study. The current study followed all procedures that complied with relevant laws and institutional guidelines and has been approved by the appropriate institutional committee. Since the study was conducted in two sessions. The second session was held after a period of a month to two months following the first session, and the participants provided written informed consent prior to the commencement of each procedure. Ethical approvals were obtained for every new session or in case of any change in the study, such as adding a new task or a new member that was joined to the study. The current study, (lab #7209), was approved by the Ethical Committee twice: May 15, 2016, and December 26, 2016. All participants provided written informed consent prior to the commencement of the procedure.

## Declaration of competing interest

The authors do not have any interest that might be interpreted as influencing the research, and APA ethical standards were followed in the study.

## Figures and Tables

**Fig. A1:**
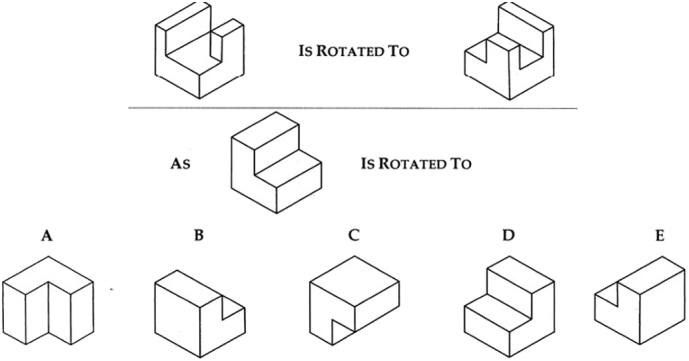
Example of an item from the Revised Purdue Spatial Visualization Test: Visualization of Rotations (revised PSVT: R) (Yoon, 2011).

**Fig. A5:**
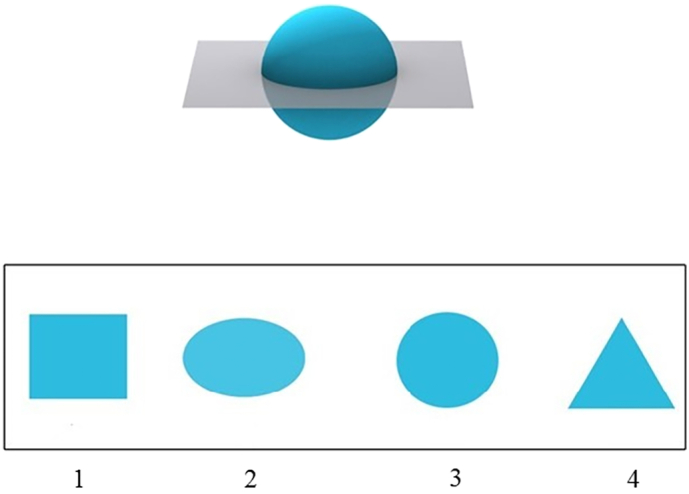
Example of an item from the Cross Section Test/Santa Barbara Solids Test (Cohen & Hegarty, 2012).

## Data Availability

I have shared the link to my data

## Footnotes

1 Task difficulty might generate another possible interpretation of the results, namely, that the easy and difficult tests form different factors according to difficulty levels. That is, easy levels constitute one factor whereas more difficult stages load on another factor. To test this possibility, we attempted to differentiate between levels of difficulty across tasks, that is, using easy levels of different tasks to load on one factor and difficult levels on another factor. However, our findings demonstrated that the measures did not load by this categorization, so it cannot be concluded that the chosen tasks reflect levels of difficulty, rather than visual-spatial components (Lohman, 1986).

2 A second-order model is constructed through a theory driven analysis. As such, it is important to note that fit indexes are not the main criteria for a good model (Brown, 2015; Byrne, 2010; Hair et al., 2009; Keith, 2014). Therefore, if our assumption is that the correlations among the first-order factors can be explained by a higher-order factor, it is important to perform a higher-order analysis.

3 A second-order model would be acceptable and even valuable, in order to address the rather high first-order factor correlations. One option to deal with the just-identified model is to constrain two parameters to be equal, which will increase the degrees of freedom. The ‘critical ratio for differences’ is a list of the differences between any two parameters. If the critical ratio is <1.96, we can assume that the two parameters are equal (see Byrne, 2010). Using this strategy, we would ‘gain’ one degree of freedom, which would allow us now to compare between the two models (first- and second-order models). Therefore, if our assumption is that the correlations among the first-order factors can be explained by a higher-order factor, it is important to perform a higher-order analysis.
